# 2832. Gepotidacin Efficacy in *E. coli* Drug-Resistant Phenotypes: A Pooled Analysis of the EAGLE-2 and EAGLE-3 Randomized Controlled Trials in Uncomplicated Urinary Tract Infection

**DOI:** 10.1093/ofid/ofad500.2442

**Published:** 2023-11-27

**Authors:** Thomas M Hooton, Caroline R Perry, Salim Janmohamed, Amanda Sheets, Jeremy Dennison, Helen Millns, Emily Jarvis, Nicole E Scangarella-Oman, Chun Huang

**Affiliations:** University of Miami, Miami, Florida, USA, Coral Gables, FL; GSK, Collegeville, PA, USA, Collegeville, Pennsylvania; GSK, Brentford, UK, Brentford, England, United Kingdom; GSK, Collegeville, PA, USA, Collegeville, Pennsylvania; GSK, Brentford, UK, Brentford, England, United Kingdom; GSK, Stevenage, UK, Stevenage, England, United Kingdom; GSK, Stevenage, UK, Stevenage, England, United Kingdom; GlaxoSmithKline plc., Collegeville, Pennsylvania; GSK, Collegeville, PA, USA, Collegeville, Pennsylvania

## Abstract

**Background:**

Uncomplicated urinary tract infections (uUTI) are among the most common community-acquired infections in women worldwide. Recommended treatment is largely empiric. Rates of antimicrobial resistance among *Escherichia coli* (*E. coli*) isolates, specifically extended-spectrum beta-lactamase positive (ESBL+) and multidrug-resistant (MDR) strains, are increasing worldwide. Gepotidacin is a first-in-class, triazaacenaphthylene, bactericidal antibiotic that inhibits bacterial DNA replication by inhibiting two enzymes, where a target-specific single mutation does not significantly impact susceptibility. We report gepotidacin efficacy against *E. coli* drug-resistant phenotypes in a pooled analysis of the EAGLE-2 and EAGLE-3 trials in uUTI.

**Methods:**

EAGLE-2 and EAGLE-3 were near-identical global, Phase 3, randomized, double-blind, double-dummy, active-controlled noninferiority trials comparing gepotidacin with nitrofurantoin. Females aged ≥ 12 years with ≥ 2 UTI symptoms were eligible and were randomized 1:1 to oral gepotidacin (1500mg) or nitrofurantoin (100mg), twice daily for 5 days. Therapeutic success at test-of-cure visit (Day 10–13) was defined as combined clinical success (complete symptom resolution) and microbiological success (from ≥ 10^5^ to < 10^3^ CFU/mL) without the need for other systemic antimicrobials. Analysis of the pooled microbiological intent-to-treat (micro-ITT) population was performed.

**Results:**

The pooled micro-ITT population comprised 1421 patients (732 gepotidacin, 689 nitrofurantoin). **Table 1** shows E. coli drug-resistant phenotypes at baseline. Therapeutic, clinical and microbiological success rates at test-of-cure numerically favored gepotidacin over nitrofurantoin for clinically important *E. coli* drug-resistant phenotypes: ESBL+, fluroquinolone-resistant (FQ-R), trimethoprim/sulfamethoxazole-resistant (SXT-R), and MDR (**Figure 1**). Adverse events were reported in 35% (gepotidacin) and 23% (nitrofurantoin) of patients.

**Figure:**
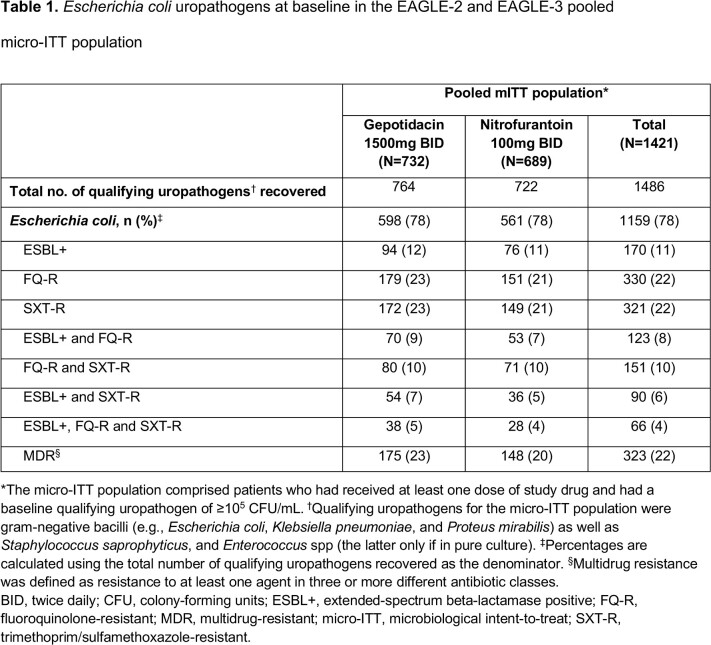

**Conclusion:**

Gepotidacin showed consistent efficacy (therapeutic, clinical and microbiological success) versus nitrofurantoin in patients with *E. coli* drug-resistant phenotypes (ESBL+, FQ-R, SXT-R, MDR). Gepotidacin has potential as a novel oral treatment for uUTI in key patient subgroups.

**Figure:**
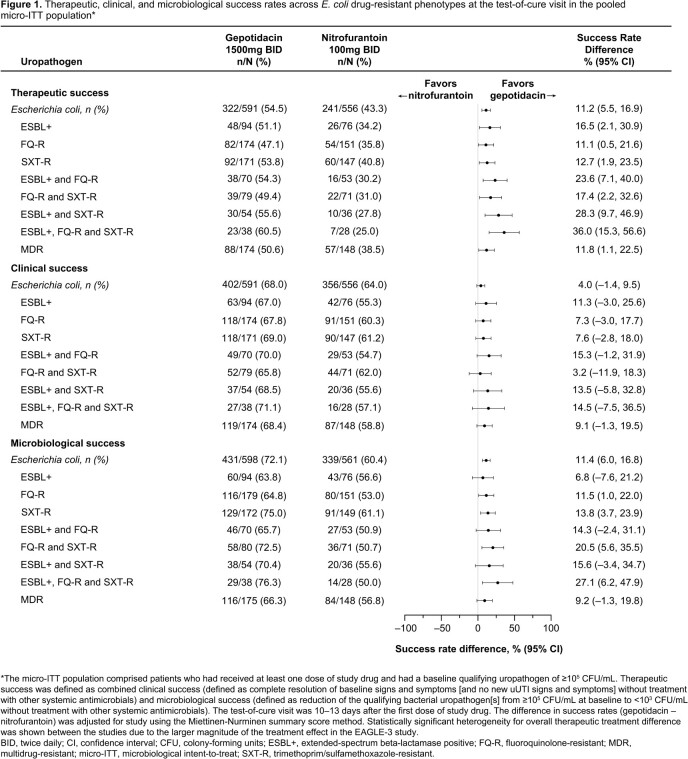

**Disclosures:**

**Thomas M. Hooton, MD**, GSK: Advisor/Consultant **Caroline R. Perry, PhD**, GSK: Employee and shareholder **Salim Janmohamed, MD**, GSK: Employee and shareholder **Amanda Sheets, PhD**, GSK: Employee and shareholder **Jeremy Dennison, MD**, GSK: Employee and shareholder **Helen Millns, PhD**, GSK: Employee and shareholder **Emily Jarvis, MSc**, GSK: Employee and shareholder **Nicole E. Scangarella-Oman, MS**, GSK: Employee and shareholder **Chun Huang, PhD**, GSK: Employee and shareholder

